# Natal habitat preference induction in large mammals—Like mother, like child?

**DOI:** 10.1002/ece3.4685

**Published:** 2018-12-11

**Authors:** Benjamin Larue, Steeve D. Côté, Martin‐Hugues St‐Laurent, Christian Dussault, Mathieu Leblond

**Affiliations:** ^1^ Département de biologie Université Laval Québec Québec Canada; ^2^ Département de biologie and Centre for Northern Studies Université Laval Québec Québec Canada; ^3^ Département de biologie, chimie et géographie, Centre for Northern Studies, and Centre for Forest Research Université du Québec à Rimouski Rimouski Québec Canada; ^4^ Direction de l’expertise sur la faune terrestre, l’herpétofaune et l’avifaune Ministère des Forêts, de la Faune et des Parcs Québec Québec Canada; ^5^ Present address: Département de biologie Université de Sherbrooke Sherbrooke Québec Canada; ^6^ Present address: Environment and Climate Change Canada, Science and Technology Branch Ottawa Ontario Canada

**Keywords:** natal experience, natal habitat preference induction, *Rangifer tarandus caribou*, repeatability, resource selection functions, woodland caribou

## Abstract

Habitat selection has received considerable attention from ecologists during the last decades, yet the underlying forces shaping individual differences in habitat selection are poorly documented. Some of these differences could be explained by the early experience of individuals in their natal habitat. By selecting habitat attributes like those encountered early in life, individuals could improve resource acquisition, survival, and ultimately fitness. This behavior, known as natal habitat preference induction (NHPI), could be particularly common in large mammals, because offspring generally stay with their mother for an extended period. We used three complementary approaches to assess NHPI in a marked population of woodland caribou (*Rangifer tarandus caribou*): (a) population‐based resource selection functions (RSFs), (b) individual‐based RSFs, and (c) behavioral repeatability analyses. All approaches compared the behavior of calves in their natal range to their behavior as independent subadults during the snow‐covered (Dec–Apr) and snow‐free (May–Nov) seasons. Using RSFs, we found that the magnitude of habitat selection between calf and subadult stages differed for most covariates, yet the signs of statistically significant effects (selection vs. avoidance) were generally the same. We also found that some habitat selection tactics were highly repeatable across life stages. Notably, caribou responses to habitat disturbances were highly repeatable year‐round, meaning that different individuals reacted differently, but consistently, to disturbances. This study highlights the potential role of natal habitat preference induction in shaping individual differences in habitat selection in large mammals and provides valuable knowledge for the management and conservation of a threatened species.

## 1 INTRODUCTION

Early life experience could shape individual differences in adult behavior and thus have major ecological and evolutionary implications (Immelmann, 1975). Notably, experience with filial or environmental stimuli may induce subsequent preference for these stimuli (Dethier, 1982). Well‐known examples of induced preference are the reactions of newborns to their presumed parents (Lorenz, 1935) or the search image acquired by predators after successfully capturing a prey (Ishii & Shimada, 2010). Induced preference for natal habitat, hereafter referred to as natal habitat preference induction (NHPI), occurs when habitat attributes encountered by an individual in its natal habitat increase the likelihood that it will select similar attributes as an adult (Davis & Stamps, 2004). NHPI could thus shape individual differences in habitat selection, or habitat selection “personalities” (Leclerc et al., 2016; Stamps & Groothuis, 2010).

Individual variability is increasingly considered in animal behavior and life‐history studies (Hamel et al., 2018; Réale, Reader, Sol, McDougall, & Dingemanse, 2007; Stamps, Briffa, & Biro, 2012). For example, studies have measured the temporal stability of individual differences in habitat selection patterns (Leclerc et al., 2016), or have linked individual differences in habitat selection (Leclerc, Dussault, & St‐Laurent, 2014; McLoughlin, Boyce, Coulson, & Clutton‐Brock, 2006) and space‐use patterns (Lafontaine, Drapeau, Fortin, & St‐Laurent, 2017) to individual differences in life‐history traits. Other studies have exposed the pitfalls of traditional population‐based habitat selection analyses that do not account for individual variability (Lesmerises & St‐Laurent, 2017). Although the importance of individual variability in habitat selection and its effects on life history are increasingly acknowledged, the potential forces that shape this variability, such as NHPI, remain poorly understood (Stamps & Groothuis, 2010).

Empirical support for NHPI originates from laboratory experiments on insects, and more recently from studies on wild birds and small mammals (reviewed by Davis & Stamps, 2004). The growing theoretical and empirical evidence for NHPI suggests that it could be an important source of individual variability in habitat selection. NHPI could be particularly common in large mammals, for which the natal period, that is, the period between birth and independence from the mother, is typically long (Ralls, Kranz, & Lundrigan, 1986). This long natal period could favor the evolution of NHPI if individuals were able to adapt their phenotype to natal‐like habitat attributes, a phenomenon referred to as adaptive phenotypic plasticity (Stamps & Davis, 2006; Stamps, Krishnan, & Willits, 2009; Via et al., 1995). Alternatively, experience learned from the reactions of the mother to various habitat attributes could trigger NHPI in large mammals, especially if this experience improved decision‐making during the establishment in a new home range (Hoppitt et al., 2008; Stamps & Davis, 2006; Stamps et al., 2009).

**Figure 1:**
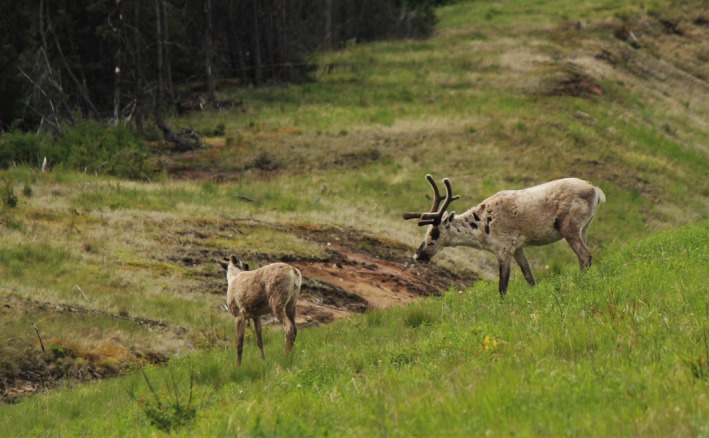
A mother with her calf, in the boreal woodland caribou (*Rangifer tarandus caribou*) population of Charlevoix, Québec, Canada. Photo credit: Benjamin Larue

The main objective of this study was to test for a potential role of NHPI in shaping individual differences in habitat selection in a sedentary population of boreal woodland caribou (*Rangifer tarandus caribou*) in Charlevoix, Québec, Canada (Figure 1). Boreal caribou usually occur in old‐growth forests providing abundant lichens, grasses, forbs, and deciduous shrubs, away from disturbed areas carrying high predation risk (such as harvested cutblocks; Rettie, Sheard, & Messier, 1997; Leblond, Dussault, Ouellet, & St‐Laurent, 2016). The availability of suitable habitat for boreal caribou has decreased across most of its distribution; however, this species is now designated as threatened under Canada's Species at Risk Act (Environment Canada, 2012). Understanding the potential mechanisms influencing postdispersal habitat selection of caribou could help identify better conservation strategies for this species.

We compared the habitat selection of caribou calves in their natal range to their selection as independent subadults using GPS telemetry. Because calves could not be equipped with GPS collars (Section 002.2 below), we used habitat selection of their mother as a proxy of their own habitat selection during their first year of life. This approach was appropriate because caribou calves are “followers” (Espmark, 1971), that is, they stay close to their mother until ~1 year of age. We hypothesized that NHPI influenced caribou habitat selection and predicted that the selection by individuals before (as calves in their natal range) versus after the separation from their mother (as independent subadults in their postdispersal range) would not differ. We also hypothesized that habitat selection would be more repeatable among life stages of a given individual than among individuals. Following this hypothesis, we predicted that variance in habitat selection coefficients among life stages would be low relative to variance among individuals. We accounted for the potential effects of seasonality by assessing habitat selection separately during the snow‐covered and snow‐free seasons, and we interpreted our results in light of varying degrees of range fidelity displayed by individuals in our study population (Lafontaine et al., 2017).

## 2 METHODS

### 2.1 Study area

**Figure 2:**
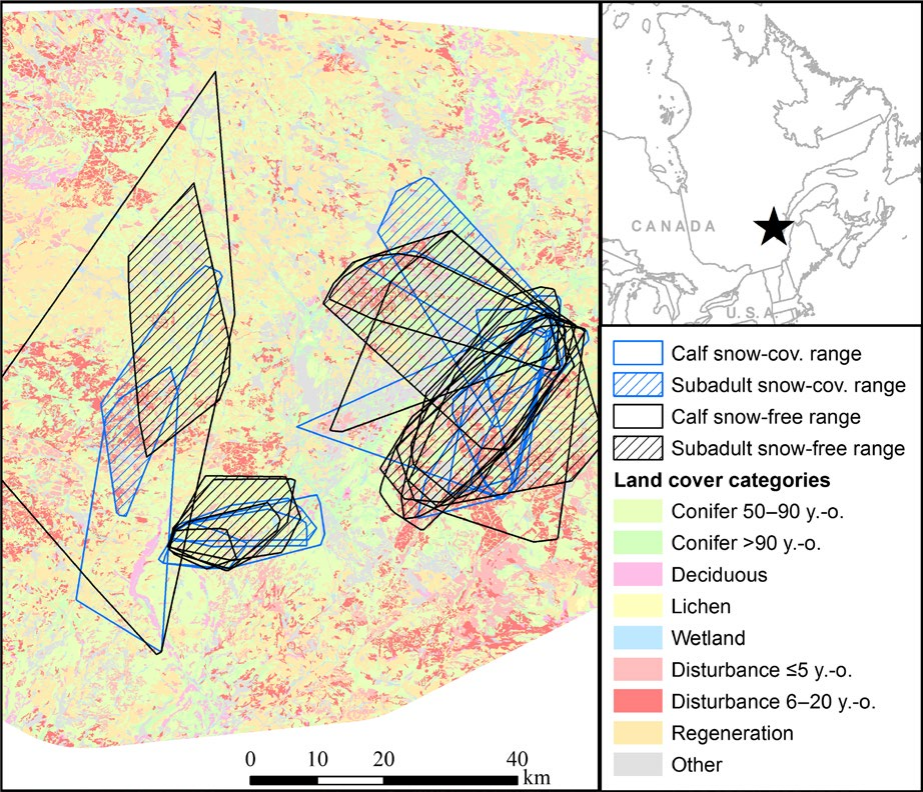
Map of the study area, showing the natal ranges of calves and the postdispersal ranges of subadults during the snow‐covered (Dec–Apr) and snow‐free seasons (May–Nov) used to study natal habitat preference induction in a boreal population of woodland caribou in Charlevoix, Québec, Canada, 2004‒2011

The study area (7,250 km^2^) was at the southern fringe of the boreal forest, in the Charlevoix region of Québec, Canada (Figure 2). It included Grands–Jardins National Park, as well as portions of Hautes‐Gorges‐de‐la‐Rivière‐Malbaie National Park, Jacques‐Cartier National Park, and Laurentides Wildlife Reserve. Climate and vegetation varied along an altitudinal gradient (400–1,100 m). At low elevation, vegetation was dominated by balsam fir (*Abies balsamea*) and yellow birch (*Betula alleghaniensis*) and, at high elevation, by black spruce (*Picea mariana*) and balsam fir. The Grands–Jardins National Park also included several open lichen woodlands. Caribou hunting was prohibited throughout the study, and logging was permitted outside national parks. Caribou were reintroduced in the Charlevoix region by the Québec government in the late 1960s (St‐Laurent & Dussault, 2012). Two aerial surveys of the study area in 2004 and 2008 estimated caribou abundance at 70 and 84 individuals, respectively (St‐Laurent & Dussault, 2012).

### 2.2 Caribou capture and telemetry

Between April 2004 and March 2008, we captured 27 adult females using a net‐gun fired from a helicopter (Potvin & Breton, 1988) and equipped them with GPS telemetry collars (models TGW‐3600 or TGW‐4600, Telonics Inc., Mesa, AZ, USA). Some of these individuals had been equipped with VHF collars during a previous study (Sebbane, Courtois, & Jolicoeur, 2008). Depending on model and year, we programmed GPS collars to record a location every 2, 3, 5, or 7 hr. Every 1 or 2 years, we recaptured individuals to download telemetry data and replace batteries. Individuals were monitored until March 2012, when collars were programmed to drop using an automated release mechanism.

Between spring 2004 and spring 2007, we located pregnant females by helicopter every 1–3 days during the calving period, looking for the presence of a calf (details in Pinard, Dussault, Ouellet, Fortin, & Courtois, 2012). These regular surveys allowed us to identify and capture 55 calves soon after birth (see Appendix S1: Table A1). GPS collars were too heavy to be placed onto newborn calves (1,300 g). Instead, we fitted calves with a 15‐g ear‐tag VHF transmitter (Holohil Al‐2C, Carp, Ontario, Canada) or an expandable 400‐g VHF collar (model M2510B; Advanced Telemetry Systems, Isanti, MN, USA) equipped with mortality sensors, with the intent of recapturing them at the subadult stage. Of the 55 calves captured, seven died from unknown causes, one from drowning, and 19 were killed by predators during the first 5 weeks after birth (Pinard et al., 2012). We lost track of eight additional calves due to transmitter/collar defects. We recaptured the remaining 20 individuals at 2.1 ± 1.6 years (mean ± *SD*) and equipped 15 of them with a GPS collar.

Caribou capture and handling procedures were approved by the animal care committees of the Ministère des Forêts, de la Faune et des Parcs du Québec, and the Université du Québec à Rimouski (certificates renewed each year: CPA # 04‐00‐02 to CPA # 10‐00‐02) based on the Canadian Council on Animal Care guidelines. Captures were performed without the use of anesthetic by experienced field personnel. Manipulations lasted on average 20 min and never more than 30 min to minimize stress on the animals.

### 2.3 Life stages and seasons

In all analyses, we used the locations of a mother accompanied by her calf as a proxy for the location of her calf during its first year of life. We then used the locations from the same offspring during its first complete year of GPS monitoring to evaluate its behavior as a subadult. During the calf stage, individuals followed their mother everywhere and occupied their natal range. During the subadult stage, individuals were independent from their mother and occupied their postdispersal range. Calf and subadult life stages were separated by a period of 460 ± 198 days (min = 239; max = 670) during which we did not know the location of the individual. We considered two seasons based on snow‐cover data from the Forêt Montmorency weather station in our study area (www.mddelcc.gouv.qc.ca/climat/donnees/). The snow‐covered season went from 1 December to 30 April, and the snow‐free season went from 1 May to 30 November.

### 2.4 Range fidelity

We evaluated the range fidelity exhibited by caribou in our study area, that is, the tendency for individuals to reuse the natal habitat after the natal period. This step was necessary because our habitat selection analyses could not distinguish between animals using the same area across life stages (range fidelity) and animals displaying similar habitat selection tactics across life stages but in different parts of the study area (NHPI). For the purposes of fidelity analyses only, we estimated individual seasonal home ranges using 99% Brownian bridge movement models (BBMM; Horne, Garton, Krone, & Lewis, 2007) with the *BBMM* package (Nielson, Sawyer, & McDonald, 2013) in R 3.4.1 (R Core Team, 2017). We estimated BBMMs using a conservative location error of 30 m and a cell size of 50 × 50 m (Sawyer, Kauffman, Nielson, & Horne, 2009). To determine seasonal range fidelity, we calculated the percentage of the postdispersal range overlapping the natal range using:

$$\frac{\text{Overlap}\left(N,D\right)}{D}\;\times \;100$$

where *N* was the area of the natal range, *D* was the area of the postdispersal range, and Overlap*(N*,* D)* was the common area between *N* and *D*. We estimated the area of overlap in ArcGIS 10.3.1 (ESRI Inc., Redlands, CA, USA).

### 2.5 Habitat variables

We used digital forest maps provided by the Québec provincial government and updated them annually to include new cutblocks, roads, and natural disturbances (fires, insect outbreaks, and windfalls). From these maps, we generated nine land cover categories based on vegetation characteristics and their importance for caribou (Courbin, Fortin, Dussault, & Courtois, 2009; Leblond et al., 2011): 50–90‐year‐old conifer‐dominated forests (including mixed‐forest stands, covering 32.0% of the study area), >90‐year‐old conifer‐dominated forests (12.5%), >50‐year‐old deciduous forests (2.1%), open lichen woodlands (1.2%), wetlands (e.g., lakes, peatlands, bogs; 2.3%), ≤5‐year‐old cutblocks and natural disturbances (fires, insect outbreaks, and windfalls; 5.6%), 6–20‐year‐old cutblocks and natural disturbances (10.4%), 21–50‐year‐old regenerating stands (originating from logging activities or natural disturbances; 25.3%), and others (e.g., human infrastructure; powerlines, unproductive open environments; 8.6%). We determined topography (i.e., elevation and slope) using an 80 × 80 m digital elevation model. Following Leblond et al. (2011), we divided roads into two categories that represented different levels of anthropogenic disturbance for caribou: active roads (paved and first‐order forestry roads) and derelict roads (second‐ and third‐order forestry roads). We centered and reduced topography and road variables to allow model convergence.

### 2.6 Population‐ and individual‐based resource selection functions

To assess habitat selection, we imported all GPS locations into ArcGIS 10.3.1 and associated each location to habitat attributes obtained from digital maps (see Table 1). We included all land cover types as binary variables (using 50–90‐year‐old conifer‐dominated forests as the reference category), as well as topography and distances to both road types as continuous variables in resource selection functions (RSF; Manly, McDonald, Thomas, McDonald, & Erickson, 2002). We used a binary‐dependent variable (1 = GPS location, 0 = random location) within a use‐availability design (Johnson, Nielsen, Merrill, McDonald, & Boyce, 2006). RSFs allowed us to contrast used habitat characteristics with those of an equivalent number of randomly generated locations in natal and postdispersal ranges during both seasons. Contrary to our analyses on range fidelity, we estimated ranges using 100% minimum convex polygons (MCP) with the *genmcp* tool in the *Geospatial Modeling Environment* (Beyer, 2012) for our RSF analyses (see Figure 2). We used MCPs rather than BBMMs to insure that ranges were sufficiently large to adequately represent availability (Leclerc, Dussault, & St‐Laurent, 2012).

**Table 1:** Variables used in resource selection functions used to assess NHPI in a boreal population of woodland caribou in Charlevoix, Québec, Canada, 2004‒2011

Groups of variables	Variable	Abbreviation
Land cover	50–90‐year‐old conifer‐dominated forest[Link]	Conifer 50–90 year‐old
	>90‐year‐old conifer‐dominated forest	Conifer >90 year‐old
	>50‐year‐old deciduous forest	Deciduous
	Open lichen woodland	Lichen
	Wetland	Wetland
	≤5‐year‐old cutblock and natural disturbance	Disturbance ≤5 year‐old
	6–20‐year‐old cutblock and natural disturbance	Disturbance 6–20 year‐old
	21–50‐year‐old regenerating stand	Regeneration
	Other	Other
Topography	Elevation (m; standardized)	Elevation
	Slope (°; standardized)	Slope
Roads	Distance to the nearest active road (km; standardized)	Active road
	Distance to the nearest derelict road (km; standardized)	Derelict road
Life stage	1st year of life (calf stage) versus 1st year of GPS monitoring (subadult stage)	Life stage
Random effect	Individual identity	CaribouID

We used 50–90‐year‐old conifer‐dominated forest as the reference category in all resource selection functions.

We estimated seasonal population‐based RSFs by fitting generalized linear mixed models using the *glmer* function in the *lme4* package (Bates, Mächler, Bolker, & Walker, 2015) in R. We used individual identity as a random intercept in all population‐based models to account for differences in sample size among individuals (Gillies et al., 2006). To compare the habitat selection of calves to the selection of subadults, we added a life stage variable to all models and used it in interaction with all other fixed variables in the models. For both seasons, we built a set of seven a priori candidate models (Table 2) which we ranked according to Akaike's information criterion corrected for small sample sizes (AIC_c_; Anderson & Burnham, 2002) using the *AICcmodavg* package (Mazerolle, 2017) in R.

**Table 2:** Ranking of population‐based resource selection functions comparing seasonal habitat selection by caribou calves in their natal range to selection by the same individuals as subadults in their postdispersal range, used to assess NHPI in a boreal population of woodland caribou in Charlevoix, Québec, Canada, 2004‒2011

Model composition	*k*	Snow‐covered season		Snow‐free season	
		AIC_c_	ΔAIC_c_	AIC_c_	ΔAIC_c_
Land cover + Topography + Roads	27	38,970	0	59,969	0
Land cover + Roads	23	41,461	2,491	60,256	288
Land cover + Topography	23	41,762	2,792	63,286	3,317
Land cover	19	43,039	4,069	63,626	3,657
Topography + Roads	11	43,186	4,217	66,527	6,558
Topography	7	45,628	6,658	70,016	10,047
Roads	7	48,447	9,477	67,157	7,188

Notes

Separate models were used for the snow‐covered (Dec–Apr) and snow‐free (May–Nov) seasons. All models included interactions between all fixed effects and Life stage, as well as CaribouID as a random effect. See Table 1 for a list of the variables included in each group of variables (i.e., Land cover, Topography, and Roads).

AIC_c_: Akaike's information criterion corrected for small sample sizes; ΔAIC_c_: difference in AIC_c_ compared to the most parsimonious model (ΔAIC_c_ = AIC_ci_ − AIC_cmin_);* k:* number of parameters.

To further evaluate the occurrence of NHPI in the population, we applied the most parsimonious seasonal population‐based habitat selection models to each individual (while also eliminating the random effect) using the *glm* function in R. To achieve this, we used individual RSFs (Leblond et al., 2016) which we compiled to highlight individual differences in habitat selection. Our goal was to better distinguish population responses from “filial” responses to habitat attributes and to assess whether individual responses in habitat selection were masked by populational responses (Lesmerises & St‐Laurent, 2017).

### 2.7 Behavioral repeatability

To assess how within‐individual variance in habitat selection compared to among‐individual variance, we first applied the most parsimonious seasonal population‐based habitat selection model (while removing the random effect and interactions) to each life stage of an individual at each season, therefore producing 4 RSFs per individual. We then extracted the coefficients of each individual × life stage × season and estimated the repeatability of habitat selection for each habitat covariate using linear mixed models with the *rptR* package (Stoffel, Nakagawa, & Schielzeth, 2017) in R. We determined habitat selection repeatability (*R*) using:

$$R=\frac{\sigma_{\text{caribouID}}^{2}}{\left(\sigma_{\text{caribouID}}^{2}+\sigma_{\text{life\textbackslash{},stage}}^{2}\right)}$$

where $\sigma_{\text{caribouID}}^{2}$ was the among‐individual variance and $\sigma_{\text{life\textbackslash{},stage}}^{2}$ was the within‐individual variance (or among‐life stage variance). This procedure directly assessed how within‐individual variance in habitat selection coefficients contrasted with among‐individual variance for all habitat variables included in the population‐based RSFs at each season (Nakagawa & Schielzeth, 2010). Like other statistical indices expressed on a scale from 0 to 1 (e.g., Spearman's *r*,* R* ^2^), the interpretation of repeatability values is somewhat subjective. Nevertheless, behavioral traits showing statistically significant repeatability values in the range of 0.2‒0.4 have been deemed as moderately to highly repeatable (Bell, Hankison, & Laskowski, 2009).

To avoid overestimating among‐individual variance in repeatability models (which would have overestimated repeatability), we accounted for functional responses in habitat selection (i.e., variations in habitat selection in response to changes in resource availability; Leclerc et al., 2016). We did this by adding the proportion of random locations that fell within each land cover type as a fixed effect in repeatability models. We used this proportion as a proxy of the availability of each land cover type in each range.

## 3 RESULTS

Of the 15 calves that survived their first year of life and that we were able to equip with a GPS collar, respectively, 9 and 8 could be paired to a GPS‐monitored mother during the snow‐covered and snow‐free seasons. We restricted our final sample to these individuals because they had complete GPS datasets during both life stages, which allowed the most valid comparison of habitat selection among the natal and postdispersal ranges. We could not use data from mothers that were equipped with a VHF collar or had collar defects during the first year of life of their calf. We retained a total of 44,942 GPS locations, with a mean of 1,322 ± 719 locations per individual × life stage × season.

Natal and postdispersal ranges during the snow‐covered season had respective sizes of 50 ± 29 km^2^ (mean ± *SD*) and 30 ± 21 km^2^ on average (estimated using 100% MCPs; *n* = 9). The mean percentage of overlap was 32% ± 23% and ranged between 0% and 65%. During the snow‐free season, natal and postdispersal ranges had respective sizes of 104 ± 97 km^2^ and 96 ± 37 km^2^ on average (*n* = 8). The mean percentage of overlap was 45% ± 23% (range = 26%–81%). Seasonal home ranges were thus on average more than twice as large during the snow‐free compared to the snow‐covered season, irrespective of life stage. The percentage of overlap was also larger during the snow‐free than the snow‐covered season.

### 3.1 Population‐ and individual‐based resource selection functions

For both seasons, the model that best explained habitat selection by caribou was the global model including land cover types, topography, and distances to active and derelict roads (Table 2). Using this model, we found that habitat selection by subadults differed significantly from the selection expressed by calves for nine of the 12 covariates for both the snow‐covered and snow‐free seasons, although the assortment of statistically significant variables differed between seasons (Table 3). Despite apparent differences in the magnitude of selection for most covariates, the signs (avoidance [*β* <0] vs. selection [*β* >0]) of statistically significant coefficients were the same across life stages for seven of nine variables during the snow‐covered season, and eight of nine variables during the snow‐free season (Table 3). Therefore, subadults in their postdispersal ranges tended to respectively avoid and select the attributes avoided and selected by calves in their natal ranges.

**Table 3:** Selection coefficients (*β*) and 95% confidence intervals (95% CI) of the variables included in the most parsimonious population‐based resource selection functions comparing the seasonal habitat selection of caribou calves in their natal range to the selection of the same individuals as subadults, used to assess NHPI in a boreal population of woodland caribou in Charlevoix, Québec, Canada, 2004‒2011

Variable	Snow‐covered season		Snow‐free season	
	*β*	95% CI	*β*	95% CI
Conifer >90 year‐old	−0.46	−0.60; −0.31	0.68	0.56; 0.80
Deciduous	0.02	−0.45; 0.48	1.53	1.25; 1.81
Lichen	0.78	0.50; 1.06	1.75	1.53; 1.97
Wetland	−0.64	−0.86; −0.43	0.79	0.64; 0.93
Disturbance ≤5 year‐old	0.66	0.52; 0.80	2.06	1.94; 2.18
Disturbance 6–20 year‐old	0.32	0.18; 0.45	1.38	1.23; 1.52
Regeneration	−1.14	−1.33; −0.95	−0.12	−0.26; 0.02
Other	1.32	1.05; 1.59	1.34	1.15; 1.52
Elevation	0.85	0.79; 0.90	0.27	0.22; 0.32
Slope	0.00	−0.05; 0.05	−0.04	−0.08; 0.00
Active road	−0.08	−0.13; −0.04	0.33	0.29; 0.37
Derelict road	1.12	1.05; 1.19	0.12	0.07; 0.17
Life stage	−0.96	−1.08; −0.84	−0.14	−0.25; −0.04
Life stage × Conifer >90 year‐old	**0.41**	**0.22**;** 0.60**	−0.06	−0.22; 0.09
Life stage × Deciduous	−12.82	−44.30; 18.66	**−0.62**	**−1.13**;** −0.12**
Life stage × Lichen	**1.90**	**1.57**;** 2.23**	**−0.97**	**−1.29**;** −0.65**
Life stage × Wetland	**1.08**	**0.81**;** 1.35**	**−0.39**	**−0.57**;** −0.20**
Life stage × Disturbance ≤5 year‐old	**0.88**	**0.68**;** 1.07**	0.04	−0.12; 0.20
Life stage × Disturbance 6–20 year‐old	**0.77**	**0.56**;** 0.97**	**0.24**	**0.05**;** 0.43**
Life stage × Regeneration	0.15	−0.10; 0.41	**0.32**	**0.14**;** 0.50**
Life stage × Other	**1.27**	**0.93**;** 1.61**	**1.57**	**1.32**;** 1.82**
Life stage × Elevation	**−0.17**	**−0.24**;** −0.09**	**−0.17**	**−0.23**;** −0.11**
Life stage × Slope	**0.15**	**0.08**;** 0.22**	**−0.13**	**−0.19**;** −0.08**
Life stage × Active road	0.02	−0.05; 0.09	−0.04	−0.09; 0.01
Life stage × Derelict road	**−0.31**	**−0.39**;** −0.23**	**0.50**	**0.44**;** 0.56**

Separate models were used for the snow‐covered (Dec–Apr) and snow‐free (May–Nov) seasons. The coefficients of variables not included in interactions represent selection by calves, whereas the coefficients of variables in interactions (e.g., Life stage × variable *x*) represent the difference in selection between the calf and subadult life stages. Selection by subadults is thus represented by the sum of the coefficients for variable *x* and the interaction Life stage × variable *x*. Statistically significant differences among life stages are highlighted in bold.

By applying the most parsimonious seasonal population‐based models to each individual separately, we found that habitat selection differed significantly between life stages in 60% and 61% of individual × habitat attribute combinations during the snow‐covered and snow‐free seasons, respectively (Appendix S2: Tables B1‒B2; results summarized in Table 4). However, and similarly to our population‐based models, the signs of statistically significant coefficients were the same across life stages for 72% of individual × habitat attribute combinations, irrespective of season. Additionally, in both seasons, there was evidence for distinct individual responses in habitat selection. Notably, several habitat covariates were either selected, avoided, or used in proportion to their availability depending on the individuals considered (Table 4). Thus, individual‐based RSFs highlighted potentially opposing individual and populational responses to habitat attributes.

**Table 4:** Individual Life stage ×habitat covariate effects determined using individual‐based resource selection functions comparing the habitat selection of caribou in their first year of life (i.e., calf stage) to the selection of the same caribou in their first year of GPS monitoring as subadults (i.e., subadult stage) during (a) the snow‐covered (Dec–Apr) and (b) snow‐free (May–Nov) seasons, in a boreal population of woodland caribou in Charlevoix, Québec, Canada, 2004‒2011

Variable	Comparison of habitat selection among life stages (calf vs. subadult)								
	ID 1	ID 2	ID 3	ID 4	ID 5	ID 6	ID 7	ID 8	ID 9
(a) Snow‐covered season									
Conifer >90 year‐old	A	A	A	S	S		A	A	P
Deciduous	P			P			A	P	
Lichen	S	S	P	S	P	P		P	
Wetland	A	A	A	P	P	S	P	A	P
Disturbance ≤5 year‐old	S	P	S	S	A	A	S	S	
Disturbance 6–20 year‐old	S	A		S	A	A	A	S	
Regeneration	A	A	A	P	A	A	A	A	
Other	P	S	P	P	P		S		
Elevation	S	S	S	S	S	S	A	S	S
Slope	A	S	A	P	A	P	S	A	P
Active road	S	S	S	A	S	P	A	S	P
Derelict road	A	A	A	P	A	P	A	A	A
(b) Snow‐free season									
Conifer >90 year‐old	S	S	P	A	P	A	P	S	
Deciduous	S	P	A	S				P	
Lichen	S	S	S	A	S	S		S	
Wetland	S	S	S	A	S	S	S	S	
Disturbance ≤5 year‐old	S	S	A	P	A	A	S	S	
Disturbance 6–20 year‐old	S	S	S	S	A	A	P	S	
Regeneration	S	A	A	A	P	A	S	A	
Other	S	S	S	A	S	S	S	P	
Elevation	S	S	P	S	A	S	A	A	
Slope	A	P	A	P	A	A	S	P	
Active road	A	P	A	A	P	A	A	A	
Derelict road	A	S	A	A	A	S	A	P	

Individual models were composed of the same habitat covariates as the most parsimonious population‐based resource selection functions without random effects (see Table 3). For each individual Life stage × habitat covariate, a blue overlay was used to indicate that selection statistically differed between life stages (i.e., significant interaction), and an orange overlay was used to indicate that selection did not statistically differ between life stages. Letters S, A, and P were, respectively, used to represent instances when calves selected, avoided, or used habitat in proportion to their availability. In cases when selection was similar among life stages, this letter also indicated selection by subadults.

### 3.2 Behavioral repeatability

By directly quantifying the repeatability of habitat selection between life stages while accounting for functional responses to habitat availability, we found several habitat attributes for which behavioral responses were highly repeatable across life stages (Table 5). Notably, we found high repeatability in the response of caribou to mature conifer and deciduous stands, ≤5 and 6–20‐year‐old disturbances, and the “other” category during the snow‐free season. During the snow‐covered season, only ≤5‐year‐old disturbances, active roads, and the “other” category had significant repeatability values (Table 5). Interestingly, responses toward habitat disturbances were generally more repeatable than responses toward natural habitat covariates, irrespective of season. Indeed, the mean annual (i.e., both seasons combined) repeatability for ≤5‐ and 6–20‐year‐old disturbances, the “other” category, and active roads combined was 0.45 ± 0.16. This value contrasted with a mean annual repeatability of 0.20 ± 0.29 for natural habitat characteristics (i.e., all other variables excluding derelict roads and regenerating stands).

**Table 5:** Repeatability (*R*) and 95% confidence intervals (95% CI) of habitat selection coefficients among life stages (i.e., within individuals) during the snow‐covered (Dec–Apr) and snow‐free (May–Nov) seasons in a boreal population of woodland caribou in Charlevoix, Québec, Canada, 2004‒2011

Variable	Snow‐covered season		Snow‐free season	
	*R*	95% CI	*R*	95% CI
Conifer >90 year‐old	0.00	0.00; 0.00	**0.54**	**0.00; 0.89**
Deciduous	0.00	0.00; 0.86	**0.87**	**0.43; 0.99**
Lichen	0.00	0.00; 0.69	0.10	0.00; 0.80
Wetland	0.00	0.00; 0.00	0.53	0.00; 0.89
Disturbance ≤5 year‐old	**0.71**	**0.15; 0.94**	**0.69**	**0.10; 0.93**
Disturbance 6–20 year‐old	0.00	0.00; 0.79	**0.54**	**0.00; 0.88**
Regeneration	0.00	0.00; 0.63	0.25	0.00; 0.85
Other	**0.44**	**0.00; 0.91**	**0.49**	**0.00; 0.89**
Elevation	0.28	0.00; 0.81	0.00	0.00; 0.68
Slope	0.00	0.00; 0.59	0.13	0.00; 0.76
Active road	**0.44**	**0.00; 0.86**	0.28	0.00; 0.84
Derelict road	0.00	0.00; 0.00	0.00	0.00; 0.72

Selection coefficients used in repeatability analyses were extracted from individual‐based RSFs fitted to every caribou at every life stage and season. Statistically significant repeatabilities (*p* < 0.05) are shown in bold.

**Figure 3:**
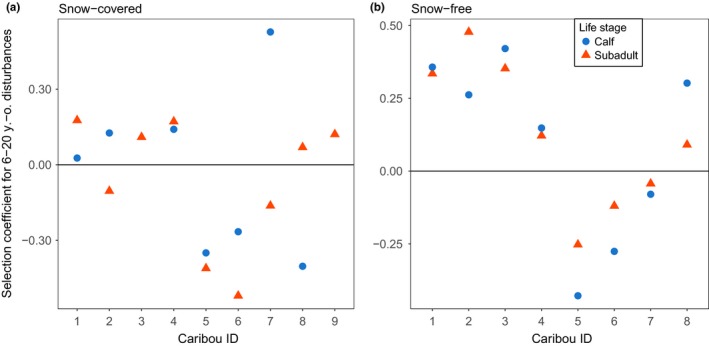
Individual selection coefficients for 6–20‐year‐old disturbances during the (a) snow‐covered (Dec–Apr) and (b) snow‐free (May – Nov) seasons in a boreal population of woodland caribou in Charlevoix, Québec, Canada, 2004‒2011. Selection coefficients were extracted from individual‐based RSFs fitted to every caribou at every life stage and season. Coefficients above and below 0 represented selection and avoidance of 6–20‐year‐old disturbances, respectively. Repeatabilities of selection coefficients for 6–20‐year‐old disturbances between individual life stages (calf =blue dots vs. subadult =orange triangles) were 0.00 and 0.54 during the snow‐covered and snow‐free seasons, respectively.

Some habitat covariates had high repeatability values because within‐individual variance in habitat selection coefficients was low relative to among‐individual variance. For instance, although three individuals avoided and five individuals selected for 6–20‐year‐old disturbances during the snow‐free season (i.e., high among‐individual variance), selection coefficients remained relatively constant from calf to subadult stages for all eight individuals (i.e., low within‐individual variance; Figure 3).

## 4 DISCUSSION

We used long‐term monitoring of individuals and habitat selection analyses to assess the occurrence of NHPI in a wild population of woodland caribou. Contrary to our prediction, the habitat selection of subadults differed statistically from the selection of calves for most habitat covariates at the population and individual levels. However, and despite substantial individual variation in selection for specific habitat attributes, most individuals displayed similar habitat selection behaviors across life stages (i.e., the signs of selection coefficients were the same across life stages in 72% of the individual × habitat attribute combinations assessed in individual‐based RSFs), which revealed qualitative support for NHPI. Repeatability analyses allowed a direct quantification of this consistency in habitat selection across life stages (Leclerc et al., 2016); we found that subadult caribou often repeated the habitat selection tactics they had used as calves. Notably, their behavioral responses toward habitat disturbances were more repeatable than their reactions toward more natural habitat features during both seasons.

### 4.1 Linking RSFs and behavioral repeatability analyses to study NHPI

Numerous studies have determined the repeatability of behavioral traits in wild animals (see Bell et al., 2009), yet the repeatability of habitat selection has seldom been assessed (but see Leclerc et al., 2016). Individual‐based RSFs allowed us to compare habitat selection among life stages and highlighted potential individual effects masked at the populational level (Lesmerises & St‐Laurent, 2017). Yet, only when we combined telemetry‐based resource selection functions with repeatability analyses were we really able to determine the proportion of variance explained by individual differences in habitat selection (Leclerc et al., 2016; Niemelä & Dingemanse, 2014). The statistically significant repeatability values of 0.44‒0.87 we obtained across several habitat covariates were indicative of a high propensity by caribou to repeat habitat selection tactics across life stages. In comparison, a review by Bell et al. (2009) on the repeatability of behavioral traits across several taxa reported repeatability values of 0.37 on average.

Among the highly repeatable behavioral traits we uncovered, the repeatability of responses to habitat disturbances by caribou seemed to be maintained year‐round. The high repeatability values for the selection of disturbed habitat indicated that different individuals reacted differently, but consistently, to disturbances. Anthropogenic disturbances represent a relatively “new” threat at the evolutionary scale, and caribou may have yet to evolve strong and consistent population responses to the cumulative effects of natural and human disturbances. Maladaptive habitat selection tactics have been suspected to lead to increased predation rates in declining caribou populations (Leech, Jelinski, DeGroot, & Kuzyk, 2017), including our study population (Dussault, Pinard, Ouellet, Courtois, & Fortin, 2012; Leblond et al., 2016). Our study provides insights as to why caribou populations may be susceptible to human development; that is, behavioral adaptation to anthropogenic disturbances is unlikely to occur quickly in caribou, if at all, because individuals tend to repeat the same habitat selection tactics across matrilines. That is, of course, only true if NHPI is maintained until females rear their own calves, an assumption that would require an even longer longitudinal dataset to assess. Still, this result highlights the importance of maintaining large caribou populations, thus insuring some degree of phenotypic diversity that would allow subsets of individuals to persist through changing environmental conditions in the long term.

The repeatability in habitat selection estimated in this study is indicative of the strength of individual responses relative to the average population response for given habitat characteristics (Nakagawa & Schielzeth, 2010). As suggested by Leclerc et al. (2016), we found that habitat selection could be a highly repeatable behavioral trait in large mammals, and we argue that NHPI could be one of the mechanisms explaining this repeatability. However, low repeatability in habitat selection for some habitat attributes is not necessarily indicative of a lack of NHPI. In fact, a strong selective pressure for a given habitat attribute could lead to the evolution of a strong population response, which would generate low among‐individual variance in habitat selection. One example of this could be the selection for open lichen woodlands, which was relatively consistent among individuals as well as among life stages within our study population. Lichens are a preferred food source for caribou (especially during winter; Rettie et al., 1997) and are scarcely distributed across the landscape. In that context, selection for lichen‐rich areas could be a favorable tactic shared by most individuals of the population, explaining its low repeatability value.

### 4.2 Range fidelity and NHPI

Range fidelity (i.e., animals using the same area across life stages) and NHPI (i.e., animals displaying similar resource selection tactics across life stages) are not mutually exclusive behaviors. For example, in our study, a subadult could have selected for the same resources it selected as a calf (i.e., similar resource selection tactics across life stages) in the same area where it once had its natal range (i.e., range fidelity). This relationship may seem obvious, yet we note that the contrary could also be true, that is, a subadult could have avoided natal‐like features in an area overlapping its natal range. This demonstrates the difference between the two concepts; NHPI and range fidelity are respective examples of habitat selection and space use, the latter being unrelated to the preference and subsequent selection of resources according to their availability (Johnson, 1980). The argument could be made, however, that habitat selection tactics are more likely to be similar in overlapping ranges than in totally separate areas. The fact that postdispersal ranges were predominantly composed of new areas relative to natal ranges (55%‒68% of ranges did not overlap, depending on seasons) supports the hypothesis that caribou were displaying NHPI and that range fidelity was unlikely to be the sole factor explaining repeatability of habitat selection in our study. Moreover, considering the large area of postdispersal ranges and the high heterogeneity of habitat attributes found in these ranges, individuals had the opportunity to display a different habitat selection tactic across life stages even when parts of their natal and postdispersal ranges overlapped.

### 4.3 NHPI as a mechanism explaining habitat selection

The potential role of NHPI in shaping habitat selection is reminiscent of the age‐old “nature versus nurture” debate (Plomin, 1994). Selection for a stimulus experienced in the natal habitat could be “innate,” that is, individuals could have a genetic predisposition to select habitat features that improve their fitness based on the genotypic legacy of their ancestors. Under this hypothesis, individual differences in habitat selection would originate from different genotypes in the population, and theory predicts that the frequency of various habitat selection tactics should be proportional to the fitness benefits provided by these differential strategies. On the other hand, habitat selection could be learned through the observation of parents, or derived from experience of the environment during early life. To determine whether preference for natal‐like habitat is inherited or induced through experience would require additional information (e.g., paternal identity and behavior; Morehouse, Graves, Mikle, & Boyce, 2016). Even then, the task of identifying all processes intervening in habitat selection would be complex, as both innate and acquired processes could act simultaneously (Pigliucci, 2001). Nevertheless, the high repeatability of habitat selection across life stages in our study population suggests that NHPI could partly explain the habitat selection tactics of boreal caribou.

### 4.4 NHPI in wildlife management and conservation

NHPI and range fidelity may benefit individuals by improving their knowledge of the habitat, allowing them to avoid predators more efficiently, or to access better food, shelter, or mates (Berteaux & Boutin, 2000; Davis & Stamps, 2004). However, in some circumstances, these behaviors may be maladaptive, partly explaining the difficulties of some species to adapt to recent environmental changes (Dussault et al., 2012; Lamb, Mowat, McLellan, Nielsen, & Boutin, 2017). In intensively managed areas where the rate of habitat alteration is high, animals exhibiting NHPI could select habitat in ways that are suited to past rather than current conditions, causing individuals to settle in poor or sink habitat (Piper, Palmer, Banfield, & Meyer, 2013). Such a response has been proposed to explain the seemingly maladaptive habitat selection tactics and subsequent poor recruitment rates of female caribou in our study population (Dussault et al., 2012; Leblond et al., 2016). In regions subjected to climate change, caribou could lag behind their optimal climatic envelope (Schloss, Nuñez, & Lawler, 2012) because of their fidelity to familiar space (Lafontaine et al., 2017). Moreover, the naivety of individuals regarding local mortality risks could impede the success of caribou translocations in areas where conditions differ from those found in the natal habitat of translocated individuals (Le Gouar, Mihoub, & Sarrazin, 2012; St‐Laurent & Dussault, 2012). We argue that the implications of early‐life habitat selection and the factors that influence this behavior, such as NHPI, should be considered in the application of wildlife management, especially for species of high conservation concern such as caribou.

## CONFLICT OF INTEREST

None declared.

## AUTHOR CONTRIBUTION

BL was responsible for analyzing data and writing the manuscript. SDC and MHSL contributed to writing the manuscript. CD conceived the project and contributed to writing the manuscript. ML contributed to conceiving the project, oversaw analyses, and contributed to writing the manuscript. All authors approved the final manuscript.

## DATA ACCESSIBILITY

Data used to perform repeatability analyses are available from the Dryad Digital Repository: https://doi.org/10.5061/dryad.24q6q70.
