# Supplementary material for: Natal habitat preference induction in large mammals—Like mother, like child?
Source: Ecol Evol. 2018 Dec 11;8(24):12629–40. doi: 10.1002/ece3.4685 (PMC6309006; doi:10.1002/ece3.4685)
Supplement: Supplementary file 1 [file ECE3-8-12629-s001.docx]

**Appendix S1.**

**Table A1.** Adult females and calves captured during the study to assess natal habitat preference induction in a boreal population of woodland caribou in Charlevoix, Québec, Canada.

| **Mother ID** | **Calf ID** | **Birth year of calf** | **GPS collar?** | **Included in analyses?** | **Reason for exclusion, if applicable^a^** |
| --- | --- | --- | --- | --- | --- |
| 07 | 07-326 | 2004 | N | N | Death in Jul/Aug 2004 (Ind) |
|  | 07-516 | 2005 | Y | N | Mother with GPS in 2008 only |
|  | 07-619 | 2006 | N | N | Collar defect |
|  | 07-709 | 2007 | Y | N | Mother with GPS in 2008 only |
| 21 | 21-321 | 2004 | N | N | Equipped with VHF |
|  | 21-502 | 2005 | Y | N | Mother with GPS in 2007 only |
|  | 21-604 | 2006 | N | N | Collar defect |
|  | 21-702 | 2007 | Y | Y |  |
| 50 | 50-512 | 2005 | N | N | Death in Jul 2005 (Pred) |
|  | 50-614 | 2006 | N | N | Death in Jun 2006 (Pred) |
|  | 50-708 | 2007 | Y | Y |  |
| 51 | 51-327B | 2004 | N | N | Collar defect |
| 52 | 52-328 | 2004 | N | N | Death in Jul/Aug 2004 (Ind) |
|  | 52-504 | 2005 | N | N | Death in Jun 2005 (Pred) |
|  | 52-601 | 2006 | Y | Y |  |
|  | 52-704 | 2007 | Y | Y |  |
| 53 | 53-322 | 2004 | N | N | Death in May 2004 (Pred) |
|  | 53-501 | 2005 | Y | Y |  |
|  | 53-602 | 2006 | N | N | Death in Jun 2006 (Pred) |
|  | 53-707 | 2007 | N | N | Death in Jun 2007 (Ind) |
| 54 | 54-ATS | 2004 | N | N | Collar defect |
| 55 | 55-330 | 2004 | N | N | Collar defect |
|  | 55-606 | 2006 | N | N | Death in Jun 2006 (Pred) |
| 57 | 57-329 | 2004 | N | N | Death in Jun 2004 (Pred) |
|  | 57-513 | 2005 | N | N | Equipped with VHF |
|  | 57-613 | 2006 | N | N | Death in Jun 2006 (Drown) |
|  | 57-711 | 2007 | N | N | Death in Jun 2007 (Ind) |
| 58 | - |  | N | N | No confirmed calving |
| 59 | 59-506 | 2005 | Y | Y |  |
|  | 59-612 | 2006 | N | N | Death in Jun 2006 (Ind) |
| 61 | 61-323 | 2004 | N | N | Death in Jun 2004 (Pred) |
| 62 | 62-327 | 2004 | N | N | Death in Jun 2004 (Pred) |
|  | 62-507 | 2005 | N | N | Death in Jun 2005 (Pred) |
|  | 62-603 | 2006 | N | N | Death in Jun 2006 (Pred) |
| 63 | - |  | N | N | No confirmed calving |
| 64 | 64-510 | 2005 | N | N | Death in Jun 2005 (Pred) |
| 65 | 65-324 | 2004 | N | N | Collar defect |
|  | 65-509 | 2005 | N | N | Death in Jun 2005 (Pred) |
| 66 | 66-615 | 2006 | N | N | Death in Jun 2006 (Pred) |
|  | 66-705 | 2007 | N | N | Death in Jun 2007 (Ind) |
| 67 | 67-508 | 2005 | N | N | Death in Jun 2005 (Pred) |
|  | 67-608 | 2006 | N | N | Death in Jun 2006 (Pred) |
| 68 | 68-519 | 2005 | N | N | Equipped with VHF |
| 69 | 69-520 | 2005 | Y | Y |  |
|  | 69-616 | 2006 | N | N | Death in Jun 2006 (Ind) |
| 70 | 70-515 | 2005 | N | N | Death in Jun 2005 (Pred) |
|  | 70-605 | 2006 | Y | Y |  |
| 71 | 71-518 | 2005 | N | N | Collar defect |
|  | 71-701 | 2007 | Y | Y | Data during one season only |
| 72 | 72-505 | 2005 | N | N | Death in Jun 2005 (Pred) |
|  | 72-610 | 2006 | N | N | Death in Jun 2006 (Pred) |
| 73 | 73-521 | 2005 | Y | N | Mother with GPS in 2006 only |
|  | 73-618 | 2006 | N | N | Equipped with VHF |
|  | 73-713 | 2007 | N | N | Equipped with VHF |
| 74 | 74-620 | 2006 | N | N | Collar defect |
| 79 | 79-714 | 2007 | Y | N | Mother with GPS in 2008 only |
| 81 | 81-714 | 2007 | Y | N | Mother with GPS in 2008 only |

^a^ Causes of death included predation (Pred), drowning (Drown), and indeterminate (Ind).
