# Supplementary material for: Natal habitat preference induction in large mammals—Like mother, like child?
Source: Ecol Evol. 2018 Dec 11;8(24):12629–40. doi: 10.1002/ece3.4685 (PMC6309006; doi:10.1002/ece3.4685)
Supplement: Supplementary file 2 [file ECE3-8-12629-s002.docx]

**Appendix S2.**

**Table B1.** Selection coefficients (β) and standard error (SE) of the variables included in individual resource selection functions comparing the habitat selection of caribou calves in their natal range to the selection of the same individuals as subadults in a boreal population of woodland caribou in Charlevoix, Québec, Canada, 2004‒2011, during the snow-covered season (Dec – Apr).

|  | **ID 1** | | | **ID 2** | | | **ID 3** | | |
| --- | --- | --- | --- | --- | --- | --- | --- | --- | --- |
| **Variable** | **β** | **SE** | **P value** | **β** | **SE** | **P value** | **β** | **SE** | **P value** |
| Conifer >90 y.-o. | -0.22 | 0.02 | 0.00 | -0.14 | 0.03 | 0.00 | -0.31 | 0.03 | 0.00 |
| Deciduous | -0.17 | 0.10 | 0.08 | -0.10 | 0.13 | 0.43 | -0.16 | 0.37 | 0.67 |
| Lichen | 0.18 | 0.04 | 0.00 | 0.32 | 0.06 | 0.00 | 0.05 | 0.04 | 0.20 |
| Wetland | -0.18 | 0.03 | 0.00 | -0.14 | 0.04 | 0.00 | -0.23 | 0.04 | 0.00 |
| Disturbance ≤5 y.-o. | 0.21 | 0.03 | 0.00 | 0.02 | 0.03 | 0.39 | 0.18 | 0.03 | 0.00 |
| Disturbance 6-20 y.-o. | 0.18 | 0.02 | 0.00 | -0.10 | 0.03 | 0.00 | 0.11 | 0.02 | 0.00 |
| Regeneration | -0.12 | 0.03 | 0.00 | -0.32 | 0.04 | 0.00 | -0.16 | 0.03 | 0.00 |
| Other | 0.04 | 0.03 | 0.23 | 0.35 | 0.09 | 0.00 | 0.04 | 0.04 | 0.26 |
| Elevation | 0.28 | 0.01 | 0.00 | 0.30 | 0.02 | 0.00 | 0.27 | 0.01 | 0.00 |
| Slope | -0.05 | 0.01 | 0.00 | 0.03 | 0.01 | 0.04 | -0.07 | 0.01 | 0.00 |
| Active road | -0.05 | 0.01 | 0.00 | -0.20 | 0.02 | 0.00 | -0.10 | 0.01 | 0.00 |
| Derelict road | 0.40 | 0.01 | 0.00 | 0.27 | 0.02 | 0.00 | 0.38 | 0.01 | 0.00 |
| Life stage | 0.09 | 0.03 | 0.00 | -0.25 | 0.06 | 0.00 | -0.33 | 0.07 | 0.00 |
| Life stage × Conifer >90 y.-o. | -0.05 | 0.04 | 0.19 | 0.12 | 0.06 | 0.05 | 0.63 | 0.07 | 0.00 |
| Life stage × Deciduous | -0.15 | 0.17 | 0.38 |  |  |  |  |  |  |
| Life stage × Lichen | 0.06 | 0.05 | 0.27 | 0.06 | 0.08 | 0.49 | 0.85 | 0.08 | 0.00 |
| Life stage × Wetland | 0.11 | 0.05 | 0.02 | 0.27 | 0.07 | 0.00 | 0.56 | 0.09 | 0.00 |
| Life stage × Disturbance ≤5 y.-o. | -0.10 | 0.04 | 0.00 | 0.55 | 0.07 | 0.00 | 0.18 | 0.08 | 0.02 |
| Life stage × Disturbance 6-20 y.-o. | -0.15 | 0.03 | 0.00 | 0.23 | 0.08 | 0.00 |  |  |  |
| Life stage × Regeneration | -0.13 | 0.05 | 0.00 | 0.31 | 0.07 | 0.00 | 0.23 | 0.08 | 0.00 |
| Life stage × Other | -0.06 | 0.06 | 0.35 | 0.16 | 0.11 | 0.14 | 0.94 | 0.08 | 0.00 |
| Life stage × Elevation | -0.14 | 0.02 | 0.00 | -0.41 | 0.03 | 0.00 | -0.23 | 0.05 | 0.00 |
| Life stage × Slope | 0.06 | 0.01 | 0.00 | -0.05 | 0.02 | 0.01 | 0.15 | 0.02 | 0.00 |
| Life stage × Active road | -0.02 | 0.01 | 0.11 | 0.25 | 0.02 | 0.00 | 0.15 | 0.03 | 0.00 |
| Life stage × Derelict road | -0.08 | 0.02 | 0.00 | -0.12 | 0.02 | 0.00 | -0.49 | 0.03 | 0.00 |

|  | **ID 4** | | | **ID 5** | | | **ID 6** | | |
| --- | --- | --- | --- | --- | --- | --- | --- | --- | --- |
| **Variable** | **β** | **SE** | **P value** | **β** | **SE** | **P value** | **β** | **SE** | **P value** |
| Conifer >90 y.-o. | 0.22 | 0.06 | 0.00 | 0.34 | 0.04 | 0.00 | 0.08 | 0.04 | 0.03 |
| Deciduous | -0.03 | 0.08 | 0.75 | 0.36 | 0.07 | 0.00 | -0.44 | 0.22 | 0.05 |
| Lichen | 0.36 | 0.07 | 0.00 | 0.11 | 0.14 | 0.45 | 0.09 | 0.11 | 0.42 |
| Wetland | 0.07 | 0.08 | 0.39 | 0.06 | 0.05 | 0.16 | 0.12 | 0.05 | 0.02 |
| Disturbance ≤5 y.-o. | 0.73 | 0.07 | 0.00 | -0.29 | 0.05 | 0.00 | -0.39 | 0.06 | 0.00 |
| Disturbance 6-20 y.-o. | 0.17 | 0.07 | 0.02 | -0.41 | 0.20 | 0.04 | -0.52 | 0.32 | 0.10 |
| Regeneration | -0.13 | 0.07 | 0.07 | -0.09 | 0.04 | 0.02 | -0.19 | 0.04 | 0.00 |
| Other | -0.17 | 0.09 | 0.06 | 0.02 | 0.16 | 0.92 | -0.47 | 0.20 | 0.02 |
| Elevation | 0.09 | 0.02 | 0.00 | 0.22 | 0.02 | 0.00 | 0.36 | 0.04 | 0.00 |
| Slope | -0.01 | 0.02 | 0.35 | -0.04 | 0.01 | 0.00 | 0.01 | 0.02 | 0.40 |
| Active road | 0.09 | 0.01 | 0.00 | -0.05 | 0.01 | 0.00 | 0.02 | 0.02 | 0.24 |
| Derelict road | -0.01 | 0.01 | 0.40 | 0.08 | 0.01 | 0.00 | -0.03 | 0.02 | 0.24 |
| Life stage | 0.20 | 0.06 | 0.00 | -0.09 | 0.03 | 0.00 | -0.31 | 0.04 | 0.00 |
| Life stage × Conifer >90 y.-o. | -0.40 | 0.07 | 0.00 | -0.29 | 0.06 | 0.00 |  |  |  |
| Life stage × Deciduous | -0.22 | 0.13 | 0.10 |  |  |  |  |  |  |
| Life stage × Lichen | -0.24 | 0.09 | 0.01 | 0.04 | 0.18 | 0.81 | 0.08 | 0.17 | 0.64 |
| Life stage × Wetland | -0.21 | 0.08 | 0.01 | -0.04 | 0.07 | 0.58 | 0.17 | 0.06 | 0.00 |
| Life stage × Disturbance ≤5 y.-o. | -0.47 | 0.07 | 0.00 | 0.12 | 0.07 | 0.09 | 0.06 | 0.07 | 0.37 |
| Life stage × Disturbance 6-20 y.-o. | -0.03 | 0.08 | 0.69 | 0.06 | 0.28 | 0.83 | 0.25 | 0.35 | 0.47 |
| Life stage × Regeneration | 0.04 | 0.08 | 0.63 | 0.00 | 0.05 | 0.98 | -0.05 | 0.05 | 0.26 |
| Life stage × Other | 0.26 | 0.10 | 0.01 | 0.04 | 0.43 | 0.93 |  |  |  |
| Life stage × Elevation | 0.16 | 0.02 | 0.00 | 0.06 | 0.03 | 0.03 | -0.14 | 0.04 | 0.00 |
| Life stage × Slope | -0.02 | 0.02 | 0.22 | 0.12 | 0.02 | 0.00 | 0.07 | 0.02 | 0.00 |
| Life stage × Active road | -0.08 | 0.02 | 0.00 | -0.01 | 0.02 | 0.49 | 0.01 | 0.02 | 0.66 |
| Life stage × Derelict road | 0.37 | 0.02 | 0.00 | -0.01 | 0.02 | 0.47 | 0.02 | 0.03 | 0.45 |

**Table B1.** (continued)

**Table B1.** (continued)

|  | **ID 7** | | | **ID 8** | | | **ID 9** | | |
| --- | --- | --- | --- | --- | --- | --- | --- | --- | --- |
| **Variable** | **β** | **SE** | **P value** | **β** | **SE** | **P value** | **β** | **SE** | **P value** |
| Conifer >90 y.-o. | -0.40 | 0.06 | 0.00 | -0.15 | 0.03 | 0.00 | -0.06 | 0.05 | 0.24 |
| Deciduous | -0.49 | 0.17 | 0.00 | -0.16 | 0.29 | 0.57 | -0.22 | 0.21 | 0.28 |
| Lichen |  |  |  | 0.00 | 0.08 | 0.98 | 0.31 | 0.10 | 0.00 |
| Wetland | -0.07 | 0.05 | 0.16 | -0.15 | 0.06 | 0.02 | -0.02 | 0.06 | 0.70 |
| Disturbance ≤5 y.-o. | 0.07 | 0.03 | 0.02 | 0.09 | 0.04 | 0.01 | -0.20 | 0.06 | 0.00 |
| Disturbance 6-20 y.-o. | -0.16 | 0.04 | 0.00 | 0.07 | 0.03 | 0.02 | 0.12 | 0.04 | 0.00 |
| Regeneration | -0.10 | 0.03 | 0.00 | -0.15 | 0.05 | 0.00 | -0.17 | 0.12 | 0.16 |
| Other | 0.44 | 0.04 | 0.00 | -0.04 | 0.05 | 0.42 | 0.07 | 0.11 | 0.52 |
| Elevation | -0.01 | 0.02 | 0.79 | 0.28 | 0.01 | 0.00 | 0.35 | 0.02 | 0.00 |
| Slope | 0.05 | 0.01 | 0.00 | -0.06 | 0.01 | 0.00 | -0.02 | 0.02 | 0.17 |
| Active road | 0.12 | 0.01 | 0.00 | -0.09 | 0.01 | 0.00 | 0.00 | 0.03 | 0.93 |
| Derelict road | 0.10 | 0.03 | 0.00 | 0.44 | 0.02 | 0.00 | 0.15 | 0.05 | 0.00 |
| Life stage | -0.04 | 0.04 | 0.35 | 0.18 | 0.05 | 0.00 | 0.30 | 0.16 | 0.05 |
| Life stage × Conifer >90 y.-o. | 0.46 | 0.07 | 0.00 | -0.04 | 0.06 | 0.44 | -0.23 | 0.10 | 0.03 |
| Life stage × Deciduous | 0.41 | 0.25 | 0.09 | -0.42 | 0.30 | 0.16 |  |  |  |
| Life stage × Lichen |  |  |  | 0.13 | 0.10 | 0.20 |  |  |  |
| Life stage × Wetland | 0.16 | 0.06 | 0.01 | -0.01 | 0.08 | 0.86 | -0.05 | 0.11 | 0.67 |
| Life stage × Disturbance ≤5 y.-o. | -0.01 | 0.05 | 0.92 | -0.13 | 0.07 | 0.06 |  |  |  |
| Life stage × Disturbance 6-20 y.-o. | 0.69 | 0.05 | 0.00 | -0.47 | 0.08 | 0.00 |  |  |  |
| Life stage × Regeneration | 0.21 | 0.04 | 0.00 | -0.32 | 0.07 | 0.00 |  |  |  |
| Life stage × Other | 0.23 | 0.05 | 0.00 |  |  |  |  |  |  |
| Life stage × Elevation | -0.04 | 0.02 | 0.07 | -0.15 | 0.02 | 0.00 | 0.22 | 0.10 | 0.02 |
| Life stage × Slope | -0.07 | 0.01 | 0.00 | 0.13 | 0.02 | 0.00 | 0.18 | 0.03 | 0.00 |
| Life stage × Active road | 0.01 | 0.01 | 0.38 | 0.01 | 0.02 | 0.58 | 0.32 | 0.08 | 0.00 |
| Life stage × Derelict road | 0.25 | 0.05 | 0.00 | -0.24 | 0.02 | 0.00 | 0.03 | 0.10 | 0.78 |

**Table B2.** Selection coefficients (β) and standard error (SE) of the variables included in individual resource selection functions comparing the habitat selection of caribou calves in their natal range to the selection of the same individuals as subadults in a boreal population of woodland caribou in Charlevoix, Québec, Canada, 2004‒2011, during the snow-free season (May – Nov).

|  | **ID 1** | | | **ID 2** | | | **ID 3** | | |
| --- | --- | --- | --- | --- | --- | --- | --- | --- | --- |
| **Variable** | **β** | **SE** | **P value** | **β** | **SE** | **P value** | **β** | **SE** | **P value** |
| Conifer >90 y.-o. | 0.26 | 0.03 | 0.00 | 0.39 | 0.03 | 0.00 | -0.04 | 0.03 | 0.11 |
| Deciduous | 0.25 | 0.05 | 0.00 | -0.22 | 0.12 | 0.07 | -0.21 | 0.14 | 0.14 |
| Lichen | 0.37 | 0.04 | 0.00 | 0.34 | 0.07 | 0.00 | 0.43 | 0.05 | 0.00 |
| Wetland | 0.30 | 0.04 | 0.00 | 0.15 | 0.04 | 0.00 | 0.14 | 0.03 | 0.00 |
| Disturbance ≤5 y.-o. | 0.60 | 0.04 | 0.00 | 0.48 | 0.03 | 0.00 | 0.53 | 0.02 | 0.00 |
| Disturbance 6-20 y.-o. | 0.36 | 0.04 | 0.00 | 0.26 | 0.04 | 0.00 | 0.42 | 0.02 | 0.00 |
| Regeneration | 0.15 | 0.04 | 0.00 | -0.14 | 0.04 | 0.00 | -0.13 | 0.03 | 0.00 |
| Other | 0.15 | 0.04 | 0.00 | 0.94 | 0.09 | 0.00 | 0.56 | 0.23 | 0.02 |
| Elevation | 0.17 | 0.01 | 0.00 | 0.14 | 0.02 | 0.00 | 0.13 | 0.01 | 0.00 |
| Slope | -0.10 | 0.01 | 0.00 | 0.01 | 0.01 | 0.28 | -0.03 | 0.01 | 0.00 |
| Active road | 0.06 | 0.01 | 0.00 | -0.02 | 0.01 | 0.07 | 0.01 | 0.01 | 0.36 |
| Derelict road | 0.05 | 0.01 | 0.00 | -0.12 | 0.02 | 0.00 | 0.13 | 0.01 | 0.00 |
| Life stage | 0.17 | 0.04 | 0.00 | 0.08 | 0.03 | 0.02 | -0.01 | 0.03 | 0.61 |
| Life stage × Conifer >90 y.-o. | -0.24 | 0.04 | 0.00 | -0.17 | 0.04 | 0.00 | 0.15 | 0.04 | 0.00 |
| Life stage × Deciduous | -0.36 | 0.12 | 0.00 | 0.12 | 0.16 | 0.46 | 0.05 | 0.20 | 0.80 |
| Life stage × Lichen | -0.71 | 0.07 | 0.00 | -0.15 | 0.09 | 0.11 | -0.33 | 0.08 | 0.00 |
| Life stage × Wetland | -0.35 | 0.05 | 0.00 | -0.07 | 0.05 | 0.18 | 0.00 | 0.04 | 0.93 |
| Life stage × Disturbance ≤5 y.-o. | -0.56 | 0.05 | 0.00 | -0.04 | 0.04 | 0.30 | 0.00 | 0.03 | 0.94 |
| Life stage × Disturbance 6-20 y.-o. | -0.02 | 0.05 | 0.67 | 0.22 | 0.04 | 0.00 | -0.07 | 0.03 | 0.04 |
| Life stage × Regeneration | -0.07 | 0.05 | 0.14 | 0.11 | 0.05 | 0.04 | 0.08 | 0.04 | 0.07 |
| Life stage × Other | -0.02 | 0.05 | 0.66 | -0.46 | 0.10 | 0.00 | 0.00 | 0.24 | 0.99 |
| Life stage × Elevation | -0.35 | 0.02 | 0.00 | -0.01 | 0.02 | 0.68 | -0.04 | 0.01 | 0.00 |
| Life stage × Slope | 0.12 | 0.01 | 0.00 | -0.01 | 0.01 | 0.43 | 0.03 | 0.01 | 0.03 |
| Life stage × Active road | 0.09 | 0.01 | 0.00 | 0.09 | 0.01 | 0.00 | 0.04 | 0.01 | 0.00 |
| Life stage × Derelict road | 0.11 | 0.01 | 0.00 | 0.27 | 0.02 | 0.00 | -0.02 | 0.02 | 0.24 |

**Table B2.** (continued)

|  | **ID 4** | | | **ID 5** | | | **ID 6** | | |
| --- | --- | --- | --- | --- | --- | --- | --- | --- | --- |
| **Variable** | **β** | **SE** | **P value** | **β** | **SE** | **P value** | **β** | **SE** | **P value** |
| Conifer >90 y.-o. | -0.13 | 0.05 | 0.00 | -0.56 | 0.47 | 0.24 | -0.51 | 0.45 | 0.25 |
| Deciduous | 0.18 | 0.05 | 0.00 | -0.52 | 0.27 | 0.06 | -0.27 | 0.44 | 0.54 |
| Lichen | -0.23 | 0.07 | 0.00 | 0.55 | 0.06 | 0.00 | 0.24 | 0.04 | 0.00 |
| Wetland | -0.20 | 0.06 | 0.00 | 0.10 | 0.04 | 0.01 | 0.13 | 0.04 | 0.00 |
| Disturbance ≤5 y.-o. | 0.05 | 0.06 | 0.44 | -0.28 | 0.05 | 0.00 | -0.13 | 0.05 | 0.01 |
| Disturbance 6-20 y.-o. | 0.15 | 0.05 | 0.00 | -0.43 | 0.19 | 0.03 | -0.28 | 0.16 | 0.08 |
| Regeneration | -0.40 | 0.06 | 0.00 | 0.01 | 0.03 | 0.84 | -0.08 | 0.03 | 0.01 |
| Other | -0.33 | 0.07 | 0.00 | 0.64 | 0.16 | 0.00 | 0.29 | 0.14 | 0.04 |
| Elevation | 0.06 | 0.01 | 0.00 | -0.08 | 0.02 | 0.00 | 0.24 | 0.02 | 0.00 |
| Slope | -0.01 | 0.01 | 0.47 | -0.03 | 0.01 | 0.02 | -0.10 | 0.01 | 0.00 |
| Active road | 0.12 | 0.01 | 0.00 | 0.00 | 0.01 | 0.89 | 0.27 | 0.02 | 0.00 |
| Derelict road | -0.02 | 0.01 | 0.03 | 0.14 | 0.01 | 0.00 | -0.11 | 0.02 | 0.00 |
| Life stage | -0.09 | 0.05 | 0.08 | 0.24 | 0.04 | 0.00 | -0.58 | 0.04 | 0.00 |
| Life stage × Conifer >90 y.-o. | 0.17 | 0.05 | 0.00 | 0.29 | 0.48 | 0.55 | 0.30 | 0.45 | 0.51 |
| Life stage × Deciduous | 0.13 | 0.07 | 0.06 |  |  |  |  |  |  |
| Life stage × Lichen | 0.29 | 0.08 | 0.00 | -0.09 | 0.12 | 0.47 | 0.26 | 0.06 | 0.00 |
| Life stage × Wetland | 0.17 | 0.07 | 0.02 | -0.24 | 0.06 | 0.00 | -0.05 | 0.05 | 0.31 |
| Life stage × Disturbance ≤5 y.-o. | 0.31 | 0.07 | 0.00 | -0.14 | 0.08 | 0.06 | -0.12 | 0.06 | 0.05 |
| Life stage × Disturbance 6-20 y.-o. | -0.03 | 0.06 | 0.68 | 0.18 | 0.27 | 0.51 | 0.16 | 0.21 | 0.45 |
| Life stage × Regeneration | 0.38 | 0.07 | 0.00 | 0.05 | 0.05 | 0.24 | 0.08 | 0.04 | 0.03 |
| Life stage × Other | 0.26 | 0.08 | 0.00 | -0.56 | 0.27 | 0.04 | -0.09 | 0.17 | 0.59 |
| Life stage × Elevation | -0.26 | 0.02 | 0.00 | 0.15 | 0.03 | 0.00 | -0.44 | 0.03 | 0.00 |
| Life stage × Slope | 0.02 | 0.02 | 0.31 | -0.01 | 0.02 | 0.39 | 0.03 | 0.01 | 0.02 |
| Life stage × Active road | -0.03 | 0.01 | 0.05 | 0.00 | 0.02 | 0.90 | -0.14 | 0.02 | 0.00 |
| Life stage × Derelict road | 0.08 | 0.01 | 0.00 | -0.13 | 0.02 | 0.00 | 0.02 | 0.02 | 0.31 |

**Table B2.** (continued)

|  | **ID 7** | | | **ID 8** | | |
| --- | --- | --- | --- | --- | --- | --- |
| **Variable** | **β** | **SE** | **P value** | **β** | **SE** | **P value** |
| Conifer >90 y.-o. | 0.02 | 0.04 | 0.69 | 0.25 | 0.02 | 0.00 |
| Deciduous | -1.11 | 0.26 | 0.00 | -0.16 | 0.11 | 0.14 |
| Lichen | 0.89 | 0.25 | 0.00 | 0.42 | 0.04 | 0.00 |
| Wetland | 0.51 | 0.03 | 0.00 | 0.09 | 0.03 | 0.00 |
| Disturbance ≤5 y.-o. | 0.52 | 0.03 | 0.00 | 0.58 | 0.02 | 0.00 |
| Disturbance 6-20 y.-o. | -0.08 | 0.04 | 0.07 | 0.30 | 0.02 | 0.00 |
| Regeneration | 0.15 | 0.02 | 0.00 | -0.07 | 0.03 | 0.01 |
| Other | 0.89 | 0.03 | 0.00 | -0.11 | 0.08 | 0.18 |
| Elevation | -0.07 | 0.01 | 0.00 | -0.06 | 0.01 | 0.00 |
| Slope | 0.06 | 0.01 | 0.00 | 0.00 | 0.01 | 0.99 |
| Active road | 0.03 | 0.01 | 0.03 | 0.09 | 0.01 | 0.00 |
| Derelict road | 0.16 | 0.02 | 0.00 | 0.00 | 0.01 | 0.76 |
| Life stage | -0.06 | 0.03 | 0.01 | -0.10 | 0.03 | 0.00 |
| Life stage × Conifer >90 y.-o. | -0.20 | 0.06 | 0.00 | -0.18 | 0.03 | 0.00 |
| Life stage × Deciduous |  |  |  | 0.00 | 0.15 | 0.98 |
| Life stage × Lichen |  |  |  | -0.49 | 0.07 | 0.00 |
| Life stage × Wetland | -0.19 | 0.04 | 0.00 | -0.19 | 0.04 | 0.00 |
| Life stage × Disturbance ≤5 y.-o. | -0.35 | 0.04 | 0.00 | -0.34 | 0.03 | 0.00 |
| Life stage × Disturbance 6-20 y.-o. | 0.04 | 0.06 | 0.54 | -0.21 | 0.03 | 0.00 |
| Life stage × Regeneration | -0.22 | 0.03 | 0.00 | -0.06 | 0.04 | 0.15 |
| Life stage × Other | -0.17 | 0.03 | 0.00 | 0.46 | 0.09 | 0.00 |
| Life stage × Elevation | 0.05 | 0.02 | 0.00 | 0.06 | 0.01 | 0.00 |
| Life stage × Slope | -0.09 | 0.01 | 0.00 | 0.03 | 0.01 | 0.01 |
| Life stage × Active road | -0.08 | 0.02 | 0.00 | -0.30 | 0.01 | 0.00 |
| Life stage × Derelict road | -0.29 | 0.03 | 0.00 | 0.22 | 0.01 | 0.00 |
